# Association of thyroid peroxidase antibodies with the rate of first-trimester miscarriage in euthyroid women with unexplained recurrent spontaneous abortion

**DOI:** 10.3389/fendo.2022.966565

**Published:** 2022-08-31

**Authors:** Meilan Liu, Dongyan Wang, Liqiong Zhu, Jianlan Yin, Xiaohui Ji, Yilei Zhong, Yuan Gao, Jianping Zhang, Yukun Liu, Rui Zhang, Hui Chen

**Affiliations:** ^1^ Department of Obstetrics and Gynecology, Sun Yat-sen Memorial Hospital, Sun Yat-sen University, Guangzhou, China; ^2^ Center for Reproductive Genetics and Reproductive Medicine, Sun Yat-sen Memorial Hospital, Sun Yat-sen University, Guangzhou, China; ^3^ Department of Obstetrics and Gynecology, The Seventh Affiliated Hospital, Sun Yat-sen University, Shenzhen, China; ^4^ Department of Obstetrics and Gynecology, YueBei People’s Hospital, Shaoguan, China; ^5^ Key Laboratory of Malignant Tumor Gene Regulation and Target Therapy of Guangdong Higher Education Institutes, Sun Yat-sen University, Guangzhou, China

**Keywords:** thyroid peroxidase antibodies, first trimester miscarriage rate, live birth rate, unexplained recurrent spontaneous abortion, endocrine

## Abstract

**Background:**

Unexplained recurrent spontaneous abortion is a serious reproductive problem of unknown etiology. Thyroid peroxidase antibodies (TPO-Ab) may be associated with pregnancy outcomes in unexplained recurrent spontaneous abortion with normal thyroid function.

**Objective:**

This study aimed to investigate the relationship between TPO-Ab and the first trimester miscarriage rate/live birth rate in women of unexplained recurrent spontaneous abortion with normal thyroid function.

**Methods:**

We retrospectively analyzed the clinical data of 297 women who met our strict inclusion criteria, comparing the first trimester miscarriage rate/live birth rate between the TPO-Ab positive and TPO-Ab negative groups. For the same purpose, we also performed subgroup analysis.

**Results:**

Of the included women, 76 (25.6%) were TPO-Ab positive, and 221 (74.4%) were negative. First trimester miscarriage rate differed between the two groups (36.8% vs 24.0%, RR = 1.54, 95% CI: 1.05–2.24, *P* = 0.030). In the younger subgroup (<35 years) and the primary RSA subgroup, First trimester miscarriage rate was also higher in the TPO-Ab positive group (33.3% vs 19.0%, RR = 1.75, 95% CI: 1.07–2.87, *P* = 0.030; 36.5% vs 21.7%, RR = 1.69, 95% CI: 1.10–2.58, *P* = 0.020). While the live birth rate was lower in women with TPO-Ab positive, the difference did not reach statistical significance, even in the subgroup analysis.

**Conclusion:**

Our results suggest that TPO-Ab is associated with first trimester miscarriage rate in euthyroid women with unexplained recurrent spontaneous abortion.

## Introduction

Recurrent spontaneous abortion (RSA) is a serious reproductive problem (1). It affects approximately 1-5% of women of reproductive age (1). Although uterine malformations, antiphospholipid syndrome, hormonal disorders, infectious diseases, and parental chromosomal abnormalities are recognized causes of RSA, approximately 50% have an unknown etiology (2). Previous studies have shown that unexplained RSA (URSA) is associated with immune abnormalities (3–5).

Thyroid disorder is frequently associated with pregnancy failure, with thyroid autoimmunity (TAI) being the most common cause (6). TAI is defined as the presence of antithyroid antibodies (ATA), such as thyroid peroxidase antibodies (TPO-Ab) and/or thyroglobulin antibodies (TG-Ab) (7). TPO-Ab is the most common antithyroid autoantibody and the majority of women with TPO-Ab have a normal thyroid function (8). It is generally accepted that TPO-Ab positive women have a higher risk of sporadic miscarriage, preterm birth and post-pauterm thyroid disease (8–10). Women with RSA have higher levels of TPO-Ab (11–13), but the effect of TPO-Ab on the clinical outcomes of URSA is highly debated. To our knowledge, only a few studies have discussed the relationship between TPO-Ab and live birth rate (LBR) in women with RSA with normal thyroid function, and the conclusions are conflicting (14–15). About 90% of abortions occur in early pregnancy, and as we know, there is almost no study report about the effects of TPO-Ab on first trimester miscarriage rate (FTMR) in euthyroid women with URSA. In recent years, more and more guildlines have defined RSA as two or more pregnancy losses, as the result, more women will be affected by RSA. Whether TPO-Ab can effectively predict pregnancy outcomes of URSA, need more evidence. In addition, we found that more and more women with RSA were elderly pregnant women and secondary RSA, and there is no research on the relationship between their abortion and TPO-Ab. Therefore, this study aimed to determine the association between TPO-Ab and LBR and first trimester outcomes in euthyroid women with URSA.

## Materials and methods

### Study design and subjects

We retrospectively analyzed the clinical data of patients with RSA who were hospitalized in the Department of Obstetrics and Gynecology at Sun Yat-sen Memorial Hospital from January 2014 to June 2021. We defined RSA as a patient who experienced two or more pregnancy losses before 24 weeks with the same partner. URSA was defined when there was no known cause. All patients were subjected to a standardized diagnostic protocol and those diagnosed with URSA were included in this study. We collected general information about the patients, including obstetric history, age, gestation week, body mass index (BMI), the status of other autoimmune antibodies (antinuclear antibodies (ANA), anti-endometrial antibodies (AEA), antiovary antibodies (AOA), anticardiolipin antibodies (ACA), anti-beta2-glycoprotein 1 antibodies (anti-β2-GP1 Ab)), first trimester outcome and final pregnancy outcome of the current pregnancy. Patients without records of first trimester or the final pregnancy outcomes were excluded. In addition, patients with abnormal levels of thyroid stimulating hormone (TSH) were screened. All the patients had TPO-Ab test, but only 89 have TG-Ab test. Therefore, based on the status of TPO-Ab, study subjects were divided into two groups: positive and negative. The Ethics Committee of Sun Yat-sen Memorial Hospital approved the work (SYSEC-KY-KS-2022-107).

### Measurement of immunological markers

TPO-Ab was detected using a chemiluminescence assay (Siemens Healthcare Diagnostics, Inc., Berlin, Germany) with a cut-off value of 60 U/mL. An indirect immunofluorescence assay (EUROIMMUN Medical Laboratory Diagnostics Co., Ltd., Beijing, China) was used to detect ANA. AEA and AOA were detected using a qualitative ELISA (Anqun Bioengineering Co., Ltd., Shenzhen, China). Total ACA and anti-β2-GP1-Ab were measured using a commercial quantitative ELISA kit (ORGENTEC Diagnostika, Berlin, Germany). TSH levels were measured by direct chemiluminescence assays (Siemens Healthcare Diagnostics, Inc., Berlin, Germany). Both ATA and Chinese guidelines mention that 4.0mU/L can be used as the cut-off point for the upper limit of TSH in early pregnancy. But the guidelines also point out that patient with TSH level > 2.5 mU/L and < 4.0 Um/L, along with TPO-Ab positive, are recommended to treatment like Levothyroxine (LT4). In this study, the patients we included in the study group were those who were TPO-Ab positive and did not have to accept treatment like LT4. According to the guidelines, they were TPO-Ab positive with TSH level < 2.5 mU. For the same reason, TSH level in the control group must be lower than 2.5 mU/L. So we defined TSH levels >2.5 mU/L as an outlier (9).

### Outcomes and associations between TPO-Ab and other characteristics

The primary outcomes of this study were FTMR and LBR, there was no patient with still birth. In China, first trimester miscarriage was defined as pregnancy loss before 12 weeks of gestation, and live birth was considered as a live baby delivered after 28 weeks of gestation in China. We then performed a subgroup analysis according to age and RSA subtypes.

### Statistical analysis

Statistical analyses were performed using SPSS (version 20.0). Baseline parameters were presented as means plus standard deviations or numbers plus percentages. Categorical variables were compared using chi-square tests, while continuous variables were compared using t-tests. As it was a univariate outcome study, the associations between TPO-Ab and FTMR or LBR were also evaluated using the chi-square test or Fisher’s exact test. Risk ratios (RR) and 95% confidence intervals (CI) were calculated using univariate logistic regression models. *P* values of 0.05 or lower were considered statistically significant.

## Results

A total of 297 euthyroid women with URSA were included in this study. Among them, 76 (25.6%) were TPO-Ab positive, and 221 (74.4%) were TPO-Ab negative.

### Baseline characteristics

As for the baseline characteristics, no statistically significant differences were found between the two groups (**Table 1**).

**Table 1:** Comparison of baseline characteristics and autoimmune antibodies in euthyroid women with URSA.

Variables	Total (n = 297)	TPO-Ab positive (n = 76)	TPO-Ab negative (n = 221)	*P* value
**Age** (y)	32.5 ± 4.3	32.0 ± 3.9	32.7 ± 4.5	0.203
**BMI** (kg/m^2^)	21.6 ± 2.9	21.4 ± 2.9	21.7 ± 2.9	0.416
**Previous miscarriages**	3.1 ± 1.2	3.2 ± 1.2	3.1 ± 1.3	0.621
**Gestation week (d)**	36.2 ± 5.5	36.5 ± 5.7	36.1 ± 5.5	0.533
**RSA Subtype**				0.864
Primary	243 (81.8)	63 (82.9)	180 (81.4)	
Secondary	54 (18.2)	13 (17.1)	41 (18.6)	
**ANA**				1.000
+	18 (6.1)	4 (5.3)	14 (6.3)	
-	279 (93.9)	72 (94.7)	207 (93.7)	
**AEA**				0.916
+	39 (13.1)	11 (14.5)	28 (12.7)	
-	239 (80.5)	60 (78.9)	179 (81.0)	
/	19 (6.4)	5 (6.6)	14 (6.3)	
**AOA**				0.957
+	5 (1.7)	1 (1.3)	4 (1.8)	
-	273 (91.9)	70 (92.1)	203 (91.9)	
/	19 (6.4)	5 (6.6)	14 (6.3)	
**ACA**				0.264
+	4 (1.3)	3 (3.9)	1 (0.5)	
-	287 (96.6)	70 (92.1)	217 (98.2)	
/	6 (2.0)	3 (3.9)	3 (1.4)	
**Anti-β2-GP1-Ab**				0.160
+	14 (4.7)	6 (7.9)	8 (3.6)	
-	279 (93.9)	68 (89.5)	211 (95.5)	
/	4 (1.3)	2 (2.6)	2 (0.9)	

+, positive; -, negative; /, unknown.

### FTMR and LBR

The FTMR was 36.8% in the TPO-Ab positive group and 24.0% in the TPO-Ab negative group. This difference was statistically significant (RR = 1.54, 95% CI: 1.05–2.24, *P* = 0.030) (**Table 2**). The LBR was lower in the TPO-Ab positive group than in the TPO-Ab negative group (63.2% vs. 72.9%), but there was no statistically significant difference (RR = 0.87, 95% CI: 0.72–1.05, *P* = 0.110) (**Table 2**).

**Table 2:** FTMR and LBR in euthyroid women with URSA.

Outcomes	TPO-Ab positive (n = 76)	TPO-Ab negative (n = 221)	RR	95% CI	*P* value
**FTMR (%)**	36.8	24.0	1.54	1.05-2.24	0.030
**LBR (%)**	63.2	72.9	0.87	0.72-1.05	0.110

### Subgroup analysis

Further subgroup analysis was also performed by age and RSA subtypes to explore whether age and RSA subtypes affected pregnancy outcomes. In the subgroup of euthyroid women with URSA younger than 35 years old, TPO-Ab positive women had significantly higher FTMR than TPO-Ab negative women (33.3% vs 19.0%, RR = 1.75, 95% CI: 1.07–2.87, *P* = 0.030). However, there was no statistically significant difference in LBR in this subgroup. On the other hand, FTMR and LBR were not statistically significantly different between TPO-Ab positive and TPO-Ab negative women in the subgroup of euthyroid women with URSA aged 35 years or older. For primary RSA, FTMR was higher in the TPO-Ab positive group than that in the TPO-Ab negative group (36.5% vs 21.7%, RR = 1.69, 95% CI: 1.10–2.58, *P* = 0.020), while LBR was not statistically different between the two groups. Regarding secondary RSA, there were also no statistically significant differences on FTMR and LBR between the TPO-Ab positive and negative groups (**Tables 3**, **4**).

**Table 3:** Subgroup analysis of age and types of RSA for FTMR in euthyroid women with URSA.

Subgroup	FTMR (%)		RR	95% CI	*P* value
	TPO-Ab positive	TPO-Abnegative			
**Age**					
< 35 years old	33.3	19.0	1.75	1.07-2.87	0.030
≥ 35 years old	47.4	33.8	1.40	0.79-2.48	0.296
**Type of RSA**					
Primary	36.5	21.7	1.69	1.10-2.58	0.020
Secondary	38.5	34.1	1.13	0.51-2.51	1.000

**Table 4:** Subgroup analysis of age and types of RSA on LBR in euthyroid women with URSA.

Subgroup	LBR (%)		RR	95% CI	*P* value
	TPO-Ab positive	TPO-Ab negative			
**Age**					
< 35 years old	66.7	76.9	0.87	0.71-1.06	0.136
≥ 35 years old	52.6	64.9	0.81	0.51-1.28	0.326
**Type of RSA**					
Primary	63.5	75.6	0.84	0.68-1.03	0.065
Secondary	61.5	61.0	1.01	0.62-1.67	1.000

## Discussion

The TPO-Ab positivity rate is 8–14% among women of childbearing age. It is generally accepted that TPO-Ab positivity rate is higher in women with RSA, ranging from 19% to 36% (14). Guideline pointed out that TPO-Ab is associated with sporadic abortion, but there is still great uncertainty about the relationship between TPO-Ab and RSA, in especially primary or secondary URSA with different ages (9). Previous studies have not directly reported whether TPO-Ab affects FTMR in euthyroid women with URSA. Furthermore, the correlation between TPO-Ab and LBR in euthyroid patients with RSA remains controversial (14, 15). Therefore, we conducted this cross-sectional observation study to investigate the relationship between TPO-Ab and the FTMR/LBR in women of URSA with normal thyroid function, hoping to provide more data to help answer these two questions. Our results showed that TPO-Ab positive euthyroid women with URSA had a higher FTMR. No statistical differences were found as for LBR, although there was an increase trend in the negative TPO-Ab group. Furthermore, the study showed that most of the pregnancy losses in both groups occurred in the first trimester of pregnancy. In subgroup anasysis, we also found that FTMR was higher in the ≥35 years or secondary subgroup, and LBR was lower in both subgroups.

Both thyroid disorder and TAI are associated with adverse pregnancy outcomes in all trimesters (6). Studies have shown that thyroid autoantibodies, especially TPO-Ab, can lead to adverse obstetric outcomes, such as miscarriage and preterm delivery, even when thyroid function is normal (16–17). However, the relationship between TPO-Ab and LBR in women with RSA has been fraught with contradictions. Rushworth et al. found no difference in LBR in patients with URSA with or without TPO-Ab (10). Some studies considered levothyroxine (LT4) but did not find any differences (18–19). However, some studies found an increase in LBR in women treated with LT4 (15, 20–21). A recent meta-analysis found that LT4 treatment had a significant effect on LBR, implying that TPO-Ab is associated with lower LBR (14). Our results showed a trend toward increased LBR in the TPO-Ab-negative group, with no statistically significant difference. Possible mechanisms regarding TPO-Ab that causes pregnancy disorders include: 1) circulating TPO-Ab indicates subclinical dysfunction; 2) TPO-Ab can directly affect placentation; 3) it is only a marker of general immune imbalance that affects the immunological relationship between mother and fetus (22). Treatments such as LT4 can reverse the adverse effects of TPO-Ab and therefore makes no difference in LBR between the two groups. More studies are needed to discuss this issue.

This study found that TPO-Ab positive euthyroid women with URSA had higher FTMR than TPO-Ab negative women. The majority of spontaneous abortions in both groups occurred in early pregnancy. We found that while the FTMR in the TPO-Ab positive group was 36.8%, the LBR was 63.2%. No pregnancy loss occurred after the first trimester. Regarding the TPO-Ab negative group, only three cases of pregnancy loss occurred in the second trimester. This suggests that TPO-Ab may mainly affect patients in the early trimester. Other factors may contribute to the adverse outcomes rather than TPO-Ab in the second or third trimester. The review by Matalon et al. has indicated a similar point of view with our study (23). Although the exact mechanism remains unclear, it can be assumed that TPO-Ab causes early miscarriage by activating the immune system and impairing the establishment of immune tolerance during pregnancy. We should pay more attention to TPO-Ab-positive URSA with normal thyroid function in the first trimester of pregnancy and consider treatments such as LT4.

Further subgroup analysis showed that FTMR in TPO-Ab positive women was significantly higher than that of TPO-Ab negative women in the < 35 years subgroup and the primary URSA subgroup respectively, but not in the ≥ 35 years subgroup and the secondary URSA group. This suggests that TPO-Ab may predominantly affect younger women and women with primary URSA. In addition, testing for TPO-Ab in both subgroups should be recommended, and more attention should be given to TPO-Ab positive patients in both subgroups. FTMR was higher in the ≥ 35-year-old and secondary subgroups in this study, while LBR was lower. Advanced maternal age is a well-recognized risk factor for adverse pregnancy outcomes. Poor quality and quantity of eggs contribute to the low pregnancy rate and the high miscarriage rate in women over 35 years of age. A recent systematic meta-analysis reported that miscarriage rates increase when women are older than 35 years, with RR ranging from 1.52 (95% CI: 1.04–2.20) to 8.80 (95% CI: 4.73–16.73) (24). Age is also an independent factor for many pregnancy complications. Women with secondary RSA who had a history of successful pregnancy were relatively older than women with primary RSA. This could explain the higher FTMR and lower LBR in TPO-Ab positive women of ≥ 35-year-old and secondary URSA subgroups. These results give us a hint that we should identify RSA patients as early as possible and screen for causes. In addition, women should conceive and bear at an appropriate age reduce the adverse effect of advance age.

Several strengths of this study are that the characteristics of the two study groups are balanced, and further subgroup analyses were performed to take more possible details into account. For the first time, we directly discussed the relationship between TPO-Ab and FTMR, which is very valuable. These findings strongly enhance our understanding of the relation of TPO-Ab and URSA pregnancy outcomes. However, there are some limitations to the current study, including the fact that the study population was small and that it was a retrospective study. This study may have been subject to allocation, selection, and recall bias. Additionally, we did not analyze the effect of treatment on the study results due to incomplete treatment information. This may also have affected the results obtained. More large population and prospective randomized studies are needed to provide a high level of evidence.

## Conclusion

Our study suggests that TPO-Ab is associated with first trimester miscarriage rate in euthyroid women with unexplained recurrent spontaneous abortion, especially in the younger and primary subgroups of RSA. Large randomized controlled studies are needed to confirm this point of view.

## Data availability statement

The original contributions presented in the study are included in the article/supplementary material, Further inquiries can be directed to the corresponding authors.

## Ethics statement

The studies involving human participants were reviewed and approved by The Ethics Committee of Sun Yat-sen Memorial Hospital. Written informed consent for participation was not required for this study in accordance with the national legislation and the institutional requirements.

## Author contributions

HC, RZ, YL and ML designed the study. DW, ML, LZ and XJ analyzed the data and followed up with the patients. ML, JY, YZ and YG were responsible for collecting research data. JZ, RZ, YL, HC and ML enrolled the patients in the study. DW, HC, RZ, YL, and ML wrote and revised the manuscript. All authors contributed to the article and approved the submitted version.

## Funding

This study was supported by the National Key Research and Development Program of China (2019YFA0801403), the National Natural Science Foundation of China (81771660, 81741017), the Science and Technology Planning Project of Guangdong Province (2017A020214018, 2017A020214003, and 2017A030310209), the Guangdong Natural Science Foundation (2018A030313023, 2018A030313162, 2018A030310162 and 18zxxt56), the Science and Technology Planning Project of Guangzhou City Central Universities (201704020034), the Technology Innovation Strategy Fund of Guangdong Province (2018A030313737), the 5010 Project of Sun Yat-sen University (2012006), and the Science and Technology Planning Project of Guangdong Province (pdjh2020b0010).

## Acknowledgments

We thank all patients who participated in this study.

## Conflict of interest

The authors declare that the research was conducted in the absence of any commercial or financial relationships that could be construed as a potential conflict of interest.

## Publisher’s note

All claims expressed in this article are solely those of the authors and do not necessarily represent those of their affiliated organizations, or those of the publisher, the editors and the reviewers. Any product that may be evaluated in this article, or claim that may be made by its manufacturer, is not guaranteed or endorsed by the publisher.
